# osp(1|2)-trivial deformation of osp(2|2)-modules structure on the spaces of symbols $S_{d}^{2}$ of differential operators acting on the space of weighted densities $F_{d}^{2}$

**DOI:** 10.1016/j.heliyon.2024.e31660

**Published:** 2024-05-23

**Authors:** Areej A Almoneef, Meher Abdaoui, Abderraouf Ghallabi

**Affiliations:** a Department of Mathematical Sciences, College of Science, Princess Nourah bint Abdulrahman University, P.O. Box 84428, Riyadh 11671, Saudi Arabia; b Department of Mathematics, College of Sciences and Humanities-Al Quwaiiyah, Shaqra University, Saudi Arabia; c Department of Mathematics, Faculty of Sciences of Sfax, BP 802, 3038 Sfax, Tunisia

**Keywords:** 17B56, 53D55, 58H15, Cohomology, Trivial deformation, Lie superalgebra, Symbol

## Abstract

Let osp(2|2) be the orthosymplectic Lie superalgebra and osp(1|2) a Lie subalgebra of osp(2|2). In our paper, we describe the cup-product $H^{1}\vee H^{1}$, where $H^{1}:=H^{1}(\mathrm{osp}(2|2),\mathrm{osp}(1|2);D_{\lambda ,\mu }^{2})$ is the first differential osp(1|2)-relative cohomology of osp(2|2) with coefficients in $D_{\lambda ,\mu }^{2}$ and $D_{\lambda ,\mu }^{2}:=\mathrm{Ho}m_{\mathrm{diff}}(F_{\lambda }^{2},F_{\mu }^{2})$ is the space of linear differential operators acting on weighted densities. This result allows us to classify the osp(1|2)-trivial deformations of the osp(2|2)-module structure on the spaces of symbols $S_{d}^{2}$. More precisely, we compute the necessary and sufficient integrability conditions of a given infinitesimal deformation of this action. Furthermore, we prove that any formal osp(1|2)-trivial deformations of osp(2|2)-modules of symbols is equivalent to its infinitisemal part. This work is the simplest generalization of a result by Laraiedh [17].

## 1 Introduction

A smooth vector field on R is represented by the Lie algebra V(R). Considering the V(R)-action on $C^{\infty }(R)$'s 1-parameter deformation:

$$L_{Y\frac{d}{dx}}^{\lambda }(\varphi )=Y\varphi^{\prime }+\lambda Y^{\prime }\varphi ,$$

where $Y,\varphi \in C^{\infty }(R)$ and $Y^{\prime }:=\frac{dY}{dx}$. The V(R)-module structure on $C^{\infty }(R)$, which is given by $L^{\lambda }$ for a fixed *λ*, is denoted by $F_{\lambda }$. Geometrically, $F_{\lambda }=\left\{\varphi dx^{\lambda }|\varphi \in C^{\infty }(R)\right\}$ is the space of polynomial weighted densities of weight λ∈R.

Along with the natural V(R)-action represented by $L_{Y}^{\lambda ,\mu }(A)$, $D_{\lambda ,\mu }:={\mathrm{Hom}}_{\mathrm{diff}}(F_{\lambda },F_{\mu })$ represents the V(R)-module of linear differential operators. The order of differential operators naturally filters each module $D_{\lambda ,\mu }$, and the space of symbols is represented by the graded module $S_{\lambda ,\mu }:=\mathrm{gr}D_{\lambda ,\mu }$. The quotient-module $D_{\lambda ,\mu }^{k}/D_{\lambda ,\mu }^{k-1}$ is isomorphic to $F_{\mu -\lambda -k}$; the principal ‘‘primary” symbol map $\sigma_{\mathrm{pr}}$ which given by:

$$A=\sum_{i=0}^{k}a_{i}(x){\left(\frac{\partial }{\partial x}\right)}^{i}\mapsto \sigma_{\mathrm{pr}}(A)=a_{k}(x){\left(dx\right)}^{\mu -\lambda -k},$$

provides the isomorphism (see, e.g., [16]). As a result, the space $S_{\lambda ,\mu }$ can be expressed as $S_{\beta }$ since it is an V(R)-module that depends exclusively on the difference β=μ−λ, and we have

$$S_{\beta }=\overset{\infty }{\underset{k=0}{⨁}}F_{\beta -k}$$

as V(R)-modules. In the sense of Richardson–Neijenhuis [18], the space $D_{\lambda ,\mu }$ is a deformation of this space $S_{\lambda ,\mu }$ and it is not an isometric as a V(R)-module to the symbol space to which it corresponds.

Deformations of several categories of structures have played an increasingly important role in mathematics and a gain in physics during the last two decades. The objective of each of these deformation problems is to identify when all associated deformation obstructions disappear, and numerous attractive methods have been developed to determine when this occurs. The deformation theory of Lie algebras has received considerable attention.

The symmetry Lie algebra of the quantum system is typically an extension of the classical symmetry algebra (see [8]). As a result, central extensions are necessary in physics.

Richardson Neijenhuis [18], [20] first considered some general questions about the theory in 1967. Deformations of Lie superalgebras considered by Binegar [9]. Fialowski [13] extended the concept of deformations in 1988 by introducing deformations based on a complete algebra of a unique maximal ideal. Additionally and in general the concept of formal versal (or miniversal) deformation was introduced, and it was demonstrated that a versal deformation existed under certain cohomology constraints. Using this framework, Fialowski and Fuchs constructed a versal deformation [14].

Nijenhuis-Richardson asserts that the module deformation theory is inextricably linked to the determines the cohomology space. For this statement to be more specific, being presented a Lie (super)-algebra C, C-module *U* and a l subalgebra of C, then the cohomology space l-relative $H^{1}(C,l;\mathrm{End}(U))$ measure infinitesimal trivial deformations in which the action is restricted to l (l-trivial deformations), where as the impediments to extending every infinitesimal l-trivial deformation to a formal deformation are connected to $H^{2}(C,l;\mathrm{End}(U))$.

This main result has been implemented by numerous authors: In 1999, Ovsienko and Roger [19] classified the deformation of the Lie algebra of vector fields in side the Lie algebra of operators pseudodifferential on $S^{1}$. In 2002, Agrebaoui, Ammar, Lecomte and Ovsienko [1] classified the deformations multiparameter of the module of symbols of operators differential. In 2003, Agrebaoui, Nizar, Mabrouk and Ovsienko [2] classified the deformations of modules of differential forms. In 2008, Ben Ammar and Boujelbene [10] classified the deformations of $V_{P}(R)$-modules of symbols trivial on sl(2). They are proved that the conditions of integrability of the infinitesimal deformation trivial on sl(2) are necessary and sufficient. Moreover, every formal deformations of $V_{P}(R)$-modules of symbols trivial on sl(2) is equivalent to a polynomial one of degree ≤2. In 2009, Basdouri and al. [6] classified the deformations of K(1)-modules of symbols trivial on osp(1|2). They are proved that the conditions of integrability of the infinitesimal deformation trivial on $R^{1|1}$ are necessary and sufficient. Moreover, every formal deformations of K(1)-modules of symbols is equivalent to a polynomial one of degree <5. On the other hand, Ammar and Kammoun [4] classified the deformations of K(1)-modules of symbols. In 2010, Basdouri, Mabrouk, Bachir and Salem [7] classified the deformation of V(1)-modules of symbols. They are proved that the conditions of integrability of the infinitesimal deformation of the second order are necessary and sufficient. In 2012, Basdouri and Ben Ammar [5] classified the deformation of sl(2)-modules and osp(1|2)-modules of symbols. In 2018, Ben Fraj, Abdaoui and Raouafi [11] classified the deformations of K(2)-modules of symbols trivial on osp(1|2). They are proved that the conditions of integrability of the infinitesimal deformation of the second order are necessary and sufficient. Lately, in 2019, Laraiedh [17] classified the aff(1|1)-trivial deformations of aff(2|1)-modules of weighted densities on the superspace $R^{1|2}$.

In this paper, first, we describe the cup-product $H^{1}\vee H^{1}$. Second, we classify the osp(1|2)-trivial deformations of the osp(2|2)-module structure on the superspace

$$S_{\mu -\lambda }^{2}=\overset{\infty }{\underset{k=0}{⨁}}F_{\mu -\lambda -\frac{k}{2}}^{2},$$

which is super analogous to the space $S_{\beta }$. We show that any formal deformation is equivalent to its infinitesimal part.

## 2 Definitions and notations

Let $R^{1|2}$ be the superspace with coordinates $\left(x,\theta_{1},\theta_{2}\right)$ where *x* is the even indeterminate, $\theta_{1}$ and $\theta_{2}$ are odd indeterminates: $\theta_{i}\theta_{j}=-\theta_{j}\theta_{i}$, 1≤i,j≤2. Consider the standard contact structure given by the 1-form

$$\alpha_{2}=dx+\theta_{1}d\theta_{1}+\theta_{2}d\theta_{2}.$$

Consider $C^{\infty }(R^{1|2})$ through the space of functions of $R^{1|2}$. A $C^{\infty }(R^{1|2})$ function has the form:

$$\Psi (x,\theta_{1},\theta_{2})=f(x)+g(x)\theta_{1}+p(x)\theta_{2}+q(x)\theta_{1}\theta_{2}$$

where $f,g,p,q\in C^{\infty }(R)$ with g,p should be anticommuting.

Suppose $V(R^{1|2})$ is defined as follows

$$V(R^{1|2})=\left\{\Psi_{0}\partial_{x}+\Psi_{1}\partial_{1}+\Psi_{2}\partial_{2}\;|\;\;\Psi_{i}\in C^{\infty }(R^{1|2})\right\},$$

where $\partial_{x}=\frac{\partial }{\partial x}$ and $\partial_{i}=\frac{\partial }{\partial \theta_{i}}$. Consider the Lie superalgebra K(2) of contact vector fields on $C^{\infty }(R^{1|2})$:

$$K(2)=\{X\in V(R^{1|2})\;|\;\text{there exists}\;f\in C^{\infty }(R^{1|2})\;\text{such that}\;L_{X}(\alpha_{2})=f\alpha_{2}\}.$$

Any $X_{f}\in K(2)$ can be written as

$$X_{f}=f\partial_{x}-\frac{1}{2}{\left(-1\right)}^{\left|f\right|}\sum_{i=1}^{2}\eta_{i}(f)\eta_{i},\;\text{where }f\in C^{\infty }(R^{1|2})\text{ and }\eta_{i}=\partial_{i}-\theta_{i}\partial_{x}\text{.}$$

Consider the orthosymplectic Lie superalgebra as a subalgebra of K(2):

$$\mathrm{osp}(2|2)=\text{Span}\left(X_{1},\;X_{x},\;X_{x^{2}},\;X_{\theta_{1}},\;X_{\theta_{2}},\;X_{x\theta_{1}},\;X_{x\theta_{2}},\;X_{\theta_{1}\theta_{2}}\right).$$

The Lie superalgebra osp(1|2) is easily determined to be a subalgebra of osp(2|2):

$$\mathrm{osp}(1|2)=\{X_{\varphi }\in \mathrm{osp}(2|2)\;|\;\partial_{2}\varphi =0\}.$$

The space of *λ*-densities is defined by:

$$F_{\lambda }^{2}=\left\{\psi (x,\theta_{1},\theta_{2})\alpha_{2}^{\lambda }\;\;|\;\;\psi (x,\theta_{1},\theta_{2})\in C^{\infty }(R^{1|2})\right\},$$

$F_{\lambda }^{2}$ is isomorphic to $C^{\infty }(R^{1|2})$, but the Lie derivative of $\psi \alpha_{2}^{\lambda }\in F_{\lambda }^{2}$ along $\varphi :=X_{\varphi }\in K(2)$ is now:

$$L_{X_{\varphi }}^{\lambda }(\psi \alpha_{2}^{\lambda })=(L_{X_{\varphi }}(\psi )+\lambda \varphi^{\prime }\psi )\alpha_{2}^{\lambda }.$$

A differential operator on $R^{1|2}$ is an operator with the following form on $C^{\infty }(R^{1|2})$:

$$A=\sum_{k=0}^{M}\sum_{\varepsilon =(\varepsilon_{1},\varepsilon_{2})}a_{k,\varepsilon }(x,\theta_{1},\theta_{2})\partial_{x}^{k}\partial_{1}^{\varepsilon_{1}}\partial_{2}^{\varepsilon_{2}};\;\;\varepsilon_{i}=0,1;\;\;M\in N.$$

Of course any differential operator defines a linear mapping $\varphi \alpha_{2}^{\lambda }⟼(A\varphi )\alpha_{2}^{\mu }$ from $F_{\lambda }^{2}$ to $F_{\mu }^{2}$ for any λ,μ∈R, thus the space of differential operators becomes a family of K(2)-modules $D_{\lambda ,\mu }^{2}$ for the natural action

$$X_{\varphi }\cdot A=L_{X_{\varphi }}^{\mu }\circ A-{\left(-1\right)}^{\left|A||\varphi \right|}A\circ L_{X_{\varphi }}^{\lambda }.$$

Since $-\eta_{i}^{2}=\partial_{x}$, and $\partial_{i}=\eta_{i}-\theta_{i}\eta_{i}^{2}$, any $A\in D_{\lambda ,\mu }^{2}$ can be expressed in this manner

$$A(\varphi \alpha_{2}^{\lambda })=\sum_{\ell =(\ell_{1},\ell_{2})}a_{\ell }(x,\theta_{1},\theta_{2})\eta_{1}^{\ell_{1}}\eta_{2}^{\ell_{2}}(\varphi )\alpha_{2}^{\mu },$$

where $a_{\ell }(x,\theta_{1},\theta_{2})$ are arbitrary functions.

## 3 Deformation theory and cohomology

### 3.1 Cohomology of Lie superalgebra

Let C be a Lie superalgebra and I be a C-modules and let h be a subalgebra of C. Let $C^{n}(C,h;I):={\mathrm{Hom}}_{h}(\Lambda^{n}(C/h);I)$ be the space of h-relative *n*-cochains of C with values in I (see, e.g., [15]). We denoted by $\delta_{n}$ the *coboundary differential operators* of $C^{n}(C,h;I)$ with value in $C^{n+1}(C,h;I)$ that it is satisfied $\delta_{n}\circ \delta_{n-1}=0$.

By definition, $H^{n}(C,h;I)$ is the quotient space

$$H^{n}(C,h;I)=Z^{n}(C,h;I)/B^{n}(C,h;I),$$

where $Z^{n}(C,h;I)$ is the kernel of $\delta_{n}$ and that it is called the space of h-relative *n*-*cocycles* and $B^{n}(C,h;I)$ is the elements in the range of $\delta_{n-1}$ and that it is called the space of h-relative *n*-*coboundaries*. For n=1 and for all $\varphi \in C^{1}(C,h;I)$,

$$\delta (\varphi )(g,\;h):={\left(-1\right)}^{\left|g||\varphi \right|}g\cdot \varphi (h)-{\left(-1\right)}^{\left|h|(|g|+|\varphi |\right)}h\cdot \varphi (g)-\varphi ([g,\;h]),\;\text{for any}\;g,h\in C.$$

### 3.2 Deformation theory

Lie algebra homomorphism deformation theory was initially thought of using just one deformation parameter [18], [20]. Recently, there has been a lot of research done on Lie (super)algebra deformations with several parameters (see, e.g., [1], [2], [4], [7], [10], [11], [14], [19]). This section provides a summary of this theory.

Let l be a subagebra of Lie superalgebra C and let $\varphi_{0}:C⟶\mathrm{End}(U)$ be an action of Lie superalgebra C on a vector superspace *U*. The infinitesimal deformations (3.1) are typically the first to be studied when investigating C-action $\varphi_{0}$'s l-trivial deformations

(3.1) $$\varphi =\varphi_{0}+t\;\Phi ,$$

where Φ:C→End(U) represents a linear map vanishing on l and *t* is a formal parameter with |t|=|Φ|. The condition of homomorphism:

[φ(a),φ(b)]=φ([a,b]),

where a,b∈C, is satisfied in order 1 in *t* ‘‘$\varphi =\varphi_{0}+t\;\Phi$” if and only if the 1-cocycle Φ is l-relative. In other words, Φ is satisfied:

$${\left(-1\right)}^{\left|a||\Phi \right|}[\varphi_{0}(a),\Phi (b)]-{\left(-1\right)}^{\left|b|(|a|+|\Phi |\right)}[\varphi_{0}(b),\Phi (a)]-\Phi ([a,\;b])=0.$$

Furthermore, $\varphi =\varphi_{0}+t\;\Phi_{1}$ and $\varphi^{\prime }=\varphi_{0}+t\;\Phi_{2}$ are equivalents if and only if there exists $\psi \in \mathrm{End}{\left(U\right)}^{l}$ such that:

$$(\Phi_{1}-\Phi_{2})(a)={\left(-1\right)}^{\left|a||\psi \right|}[\varphi_{0}(a),\psi ]:=\delta \psi (a),$$

where *δ* defines the differential of the cochains on C with values in End(U) (see, e.g., [3], [15], [18]). Hence, up to equivalence, the cohomology space ‘‘$H^{1}(C,l;\mathrm{End}(U))$” specifies and categorizes infinitesimal deformations.

If $\dim H^{1}(g,l;\mathrm{End}(V))$=*n* and $H^{1}(C,l;\mathrm{End}(U))$=span$\left\{\Phi_{1},\ldots ,\Phi_{n}\right\}$, where $\Phi_{1},\ldots ,\Phi_{n}$ are 1-cocycles, then

$$\varphi =\varphi_{0}+\sum_{i=1}^{n}t_{i}\;\Phi_{i},$$

where the parameters $t_{i},\;i=1,\cdots n$ are independent and $\left|t_{i}|=|\Phi_{i}\right|$. This infinitesimal deformation is being attempted to be expanded into a formal one:

$$\varphi =\varphi_{0}+\sum_{i=1}^{n}t_{i}\;\Phi_{i}+\sum_{i,j}t_{i}t_{j}\;\varphi_{ij}^{\left(2\right)}+\cdots ,$$

where the maps $\varphi_{ij}^{\left(2\right)},\varphi_{ijk}^{\left(3\right)},\ldots$ define from C to value in End(U) are linear and $|\varphi_{ij}^{\left(2\right)}|=|t_{i}t_{j}|,|\varphi_{ijk}^{\left(3\right)}|=|t_{i}t_{j}t_{k}|,\ldots$ such that

[φ(a),φ(b)]=φ([a,b]),a,b∈C

It is well known that this condition causes all of the obstructions, which are lie in $H^{2}(C,l;U)$. As a result, we will make $t_{1},\ldots ,t_{n}$ and $t_{n}$ subject to more algebraic relationships. Suppose R is an ideal in $C[[t_{1},\ldots ,t_{n}]]$ generated by a set of these relations:

$$C[[t_{1},\ldots ,t_{n}]]/R$$

is a supercommutative associative superalgebra with unity.

## 4 Cohomology and deformation of $S_{\beta }^{2}$

We study the osp(1|2)-trivial deformations of the osp(2|2)-module structure on the space of symbols:

$$S_{\beta }^{2}=\overset{\infty }{\underset{k=0}{⨁}}F_{\beta -\frac{k}{2}}^{2}.$$

The infinitesimal deformations are classified by:

$$H_{\mathrm{diff}}^{1}\left(\mathrm{osp}(2|2),\mathrm{osp}(1|2);S_{\beta }^{2}\right)=\underset{i,j\geq 0}{⨁}H_{\mathrm{diff}}^{1}\left(\mathrm{osp}(2|2),\mathrm{osp}(1|2);D_{\beta -\frac{j}{2},\beta -\frac{i}{2}}^{2}\right).$$

Ben Fraj and Boujelben computations of the space $H_{\mathrm{diff}}^{1}(\mathrm{osp}(2|2),\mathrm{osp}(1|2);D_{\lambda ,\mu }^{2})$ [12], they stated the following result:

Theorem 4.1 *[12]*

$$H_{\mathrm{diff}}^{1}(\mathrm{osp}(2|2),\mathrm{osp}(1|2);D_{\lambda ,\mu }^{2})≃\left\{ \begin{array}{ccc} R\; & \text{if}\; & \mu =\lambda \neq 0\;\text{or}\;(\lambda ,\mu )=(\frac{-k}{2},\frac{k}{2})\\ 0\; & \text{otherwise}.\; \end{array}\right.$$

*Moreover, we have*

i) *If*2β∉N*, then*
  $$H_{\mathrm{diff}}^{1}\left(\mathrm{osp}(2|2),\mathrm{osp}(1|2);S_{\beta }^{2}\right)=\underset{0\leq k}{⨁}H_{\mathrm{diff}}^{1}\left(\mathrm{osp}(2|2),\mathrm{osp}(1|2);D_{\beta -\frac{k}{2},\beta -\frac{k}{2}}^{2}\right).$$
  *The space*$H_{\mathrm{diff}}^{1}\left(\mathrm{osp}(2|2),\mathrm{osp}(1|2);D_{\beta -\frac{k}{2},\beta -\frac{k}{2}}\right)$*is spanned by:*
  $$\omega_{\beta -\frac{k}{2},\beta -\frac{k}{2}}(G)=(2\beta -k){\bar{\eta }}_{1}(\partial_{2}(G))-{\left(-1\right)}^{\left|G\right|}(\partial_{2}(G){\bar{\eta }}_{1}+\theta_{2}{\bar{\eta }}_{2}{\bar{\eta }}_{1}(G){\bar{\eta }}_{2})$$
ii) *If*2β=m∈N*, then*
  $$\begin{array}{ccc} H_{\mathrm{diff}}^{1}\left(\mathrm{osp}(2|2),\mathrm{osp}(1|2);S_{\beta }^{2}\right)\; & =\; & \overset{m}{\underset{k=1}{⨁}}H_{\mathrm{diff}}^{1}\left(\mathrm{osp}(2|2),\mathrm{osp}(1|2);D_{-\frac{k}{2},\frac{k}{2}}^{2}\right)\\ \; & ⊕\; & \overset{m}{\underset{k=-\infty }{⨁}}H_{\mathrm{diff}}^{1}\left(\mathrm{osp}(2|2),\mathrm{osp}(1|2);D_{\frac{k}{2},\frac{k}{2}}^{2}\right). \end{array}$$
  *The space*$H_{\mathrm{diff}}^{1}\left(\mathrm{osp}(2|2),\mathrm{osp}(1|2);D_{\frac{k}{2},\frac{k}{2}}\right)$*is spanned by:*
  $${ϒ}_{\frac{k}{2},\frac{k}{2}}(G)=k{\bar{\eta }}_{1}(\partial_{2}(G))-{\left(-1\right)}^{\left|G\right|}(\partial_{2}(G){\bar{\eta }}_{1}+\theta_{2}{\bar{\eta }}_{2}{\bar{\eta }}_{1}(G){\bar{\eta }}_{2})$$
  *The space*$H_{\mathrm{diff}}^{1}\left(\mathrm{osp}(2|2),\mathrm{osp}(1|2);D_{-\frac{k}{2},\frac{k}{2}}\right)$*is spanned by:*
  $$\Gamma_{-\frac{k}{2},\frac{k}{2}}(G)=k{\bar{\eta }}_{1}(\partial_{2}(G)){\bar{\eta }}_{1}{\bar{\eta }}_{2}^{2k-1}-{\left(-1\right)}^{\left|G\right|}(\partial_{2}(G){\bar{\eta }}_{2}^{2k+1}-{\bar{\eta }}_{1}(\theta_{2}\partial_{2}(G)){\bar{\eta }}_{1}^{2k+1})$$

In our study, any infinitesimal osp(1|2)-trivial deformations of the osp(2|2)-module structure on $S_{\beta }^{2}$ is of the form:

(4.2) $$\overset{˜}{L}=L+L^{1}$$

where

$$L^{1}=\left\{ \begin{array}{ccc} \sum_{k\geq 0}t_{\beta -\frac{k}{2},\beta -\frac{k}{2}}\omega_{\beta -\frac{k}{2},\beta -\frac{k}{2}}\; & \text{if}\; & 2\beta \notin N\\ \sum_{k\leq m}t_{\frac{k}{2},\frac{k}{2}}{ϒ}_{\frac{k}{2},\frac{k}{2}}+\sum_{k=1}^{m}t_{\frac{k}{2},\frac{k}{2}}\Gamma_{-\frac{k}{2},\frac{k}{2}}\; & \text{if}\; & 2\beta =m\in N \end{array}\right.$$

where $t_{\beta -\frac{k}{2},\beta -\frac{k}{2}}$, $t_{\frac{k}{2},\frac{k}{2}}$ and $t_{-\frac{k}{2},\frac{k}{2}}$ are independent parameters.

Now, we will formalize the infinitesimal deformation (4.2):

$$\overset{˜}{L}=L+L^{1}+\sum_{i}Q_{i}^{2}L_{i}^{2}+\sum_{i}Q_{i}^{3}L_{i}^{3}+\cdots ,$$

where the map:

$$\overset{˜}{L}:\mathrm{osp}(2|2)⟶C[[t_{\beta -\frac{k}{2},\beta -\frac{k}{2}},t_{\frac{k}{2},\frac{k}{2}},t_{-\frac{k}{2},\frac{k}{2}}]]⊗\mathrm{End}(S_{\beta }^{2}),$$

meets the homomorphism condition,

(4.3) $${\overset{˜}{L}}_{\left[g,h\right]}=[{\overset{˜}{L}}_{g},{\overset{˜}{L}}_{h}],$$

and the higher order terms $L_{i}^{2}$, $L_{i}^{3}\cdots$ are linear maps from osp(2|2) to $\mathrm{End}(S_{\beta }^{2})$. $Q_{i}^{j}$ are monomial in terms of the parameters $t_{\beta -\frac{k}{2},\beta -\frac{k}{2}}$, $t_{\frac{k}{2},\frac{k}{2}}$, $t_{-\frac{k}{2},\frac{k}{2}}$ with degree *j* and with $\left|Q_{i}^{j}|=|L_{i}^{j}\right|$.

Setting

$$\rho =\overset{˜}{L}-L,\;\;L^{2}=\sum_{i}Q_{i}^{2}L_{i}^{2},\;\;L^{3}+\sum_{i}Q_{i}^{3}L_{i}^{3},\cdots .$$

The homomorphism condition (4.3) can be written as follows:

$$[\rho (g),L_{h}]+[L_{g},\rho (h)]-\rho ([g,h])+\sum_{i,j>0}\;[L_{g}^{i},L_{h}^{j}]=0,$$

or equivalently

(4.4) $$\delta \rho +\frac{1}{2}\rho \vee \rho =0,$$

where the differential of the chain *ρ* is represented by *δρ* and for each linear map ζ,ξ:C⟶End(U), where C is a Lie superalgebra and *U* is a vector superspace, ∨ represents the standard cup-product as described by:

$$(\zeta \vee \xi )(a,b)={\left(-1\right)}^{\left|a||\xi \right|}[\zeta (a),\xi (b)]+{\left(-1\right)}^{\left|\zeta |(|a|+|\xi |\right)}[\xi (a),\zeta (b)].$$

From (4.4) for any $L^{k}$, we can derive the following equation:

(4.5) $$\delta L^{k}+\frac{1}{2}\sum_{i+j=k}L^{i}\vee L^{j}=0.$$

The first obstruction to the integration of an infinitesimal deformation is presented by the first relation:

$$\delta L^{2}+\frac{1}{2}L^{1}\vee L^{1}=0.$$

Hence, $L^{1}\vee L^{1}$ must therefore be a coboundary.

It is evident that the bilinear map $C_{1}\vee C_{2}$ is an l-relative 2-cocycle for any two 1-cocycles $C_{1}$ and $C_{2}\in Z^{1}(C,l;\mathrm{End}(U))$. Furthermore, $C_{1}\vee C_{2}$ is a l-relative 2-coboundary ‘‘$C_{1}\vee C_{2}=\delta A$” if one of the cocycles, $C_{1}$ or $C_{2}$, is a l-relative coboundary. As a result that the operation (4.4) generates a bilinear map:

$$H^{1}(C,l;\mathrm{End}(U))⊗H^{1}(C,l;\mathrm{End}(U))⟶H^{2}(C,l;\mathrm{End}(U)).$$

All the obstructions lie in $H^{2}(C,l;\mathrm{End}(U))$ and they are in the image of $H^{1}(C,l;\mathrm{End}(U))$ under the cup-product. Therefore, the cup-product $H^{1}\vee H^{1}$ is described in the following section.

## 5 The cup-product $H^{1}\vee H^{1}$

We have to distinguish two cases:

$\underline{\text{Case 1: 2}\beta \notin N}$. In this case, we have $L^{\left(1\right)}=\sum_{k\geq 0}t_{\beta -\frac{k}{2},\beta -\frac{k}{2}}\omega_{\beta -\frac{k}{2},\beta -\frac{k}{2}}$.

Theorem 5.1 *If*2β∉N*, then, the image*$H^{1}\vee H^{1}$*of*$H_{\mathrm{diff}}^{1}\left(\mathrm{osp}(2|2),\mathrm{osp}(1|2);D_{\beta -\frac{k}{2},\beta -\frac{k}{2}}\right)$*under the cup-product is a one-dimensional subspace of*$H_{\mathrm{diff}}^{2}\left(\mathrm{osp}(2|2),\mathrm{osp}(1|2);D_{\beta -\frac{k}{2},\beta -\frac{k}{2}}\right)$*spanned by*

$$\Omega_{\beta -\frac{k}{2},\beta -\frac{k}{2}}.$$

Proof In this case, the space $H^{1}\vee H^{1}$ is generated by the cup-product:

$$\Omega_{\beta -\frac{k}{2},\beta -\frac{k}{2}}=\omega_{\beta -\frac{k}{2},\beta -\frac{k}{2}}\vee \omega_{\beta -\frac{k}{2},\beta -\frac{k}{2}}.$$

So, we have to prove that $\Omega_{\beta -\frac{k}{2},\beta -\frac{k}{2}}$ is a non-trivial osp(1|2)-relative 2-cocycle. That is, the equation

(5.6) $$\begin{array}{c} \alpha .\Omega_{\beta -\frac{k}{2},\beta -\frac{k}{2}}=\delta (c), \end{array}$$

where α∈R, $c\in C_{\mathrm{diff}}^{1}\left(\mathrm{osp}(2|2),\mathrm{osp}(1|2);D_{\beta -\frac{k}{2},\beta -\frac{k}{2}}^{2}\right)$, has a solution if and only if α=0.

First of all, we have

$$\Omega_{\beta -\frac{k}{2},\beta -\frac{k}{2}}(\phi ,\psi )=\omega_{\beta -\frac{k}{2},\beta -\frac{k}{2}}\vee \omega_{\beta -\frac{k}{2},\beta -\frac{k}{2}}(\phi ,\psi )={\left(-1\right)}^{\left|\phi ||\omega_{\beta -\frac{k}{2},\beta -\frac{k}{2}}\right|}[\omega_{\beta -\frac{k}{2},\beta -\frac{k}{2}}(\phi ),\omega_{\beta -\frac{k}{2},\beta -\frac{k}{2}}(\psi )]\;\;+{\left(-1\right)}^{\left|\omega_{\beta -\frac{k}{2},\beta -\frac{k}{2}}|(|\phi |+|\omega_{\beta -\frac{k}{2},\beta -\frac{k}{2}}|\right)}[\omega_{\beta -\frac{k}{2},\beta -\frac{k}{2}}(\phi ),\omega_{\beta -\frac{k}{2},\beta -\frac{k}{2}}(\psi )]=2[\omega_{\beta -\frac{k}{2},\beta -\frac{k}{2}}(\phi ),\omega_{\beta -\frac{k}{2},\beta -\frac{k}{2}}(\psi )]=2[(2\beta -k)\eta_{1}(\partial_{2}(\phi )),(2\beta -k)\eta_{1}(\partial_{2}(\psi ))]\;\;-2{\left(-1\right)}^{\left|\psi \right|}[(2\beta -k)\eta_{1}(\partial_{2}(\phi )),\partial_{2}(\psi )\eta_{1}+\theta_{2}\eta_{2}\eta_{1}(\psi )\eta_{2}]\;\;-2{\left(-1\right)}^{\left|\phi \right|}[\partial_{2}(\phi )\eta_{1}+\theta_{2}\eta_{2}\eta_{1}(\phi )\eta_{2},(2\beta -k)\eta_{1}(\partial_{2}(\psi ))]\;\;+2{\left(-1\right)}^{\left|\phi |+|\psi \right|}[\partial_{2}(\phi )\eta_{1}+\theta_{2}\eta_{2}\eta_{1}(\phi )\eta_{2},\partial_{2}(\psi )\eta_{1}+\theta_{2}\eta_{2}\eta_{1}(\psi )\eta_{2}]=2{\left(-1\right)}^{\left|\phi \right|}(2\beta -k)\left(\partial_{x}\partial_{2}(\phi )\partial_{2}(\psi )+\partial_{2}(\phi )\partial_{x}\partial_{2}(\psi )\right)\;\;+4{\left(-1\right)}^{\left|\phi \right|}\partial_{2}(\phi )\partial_{2}(\psi )\partial_{x}+2\theta_{1}\partial_{2}(\phi )\partial_{1}\partial_{2}(\psi )\partial_{x}\;\;\;-2{\left(-1\right)}^{\left|\phi \right|}\theta_{1}\partial_{1}\partial_{2}(\phi )\partial_{2}(\psi )\partial_{x}-2{\left(-1\right)}^{\left|\psi \right|}\eta_{1}\partial_{2}(\phi )\partial_{2}(\psi )\partial_{1}\;\;\;+2{\left(-1\right)}^{\left|\phi |+|\psi \right|}\partial_{2}(\phi )\eta_{1}\partial_{2}(\psi )\partial_{1}+2{\left(-1\right)}^{\left|\psi \right|}\theta_{2}\partial_{2}(\phi )\partial_{x}\partial_{2}(\psi )\partial_{2}\;\;\;+2{\left(-1\right)}^{\left|\psi \right|}\theta_{2}\partial_{x}\partial_{2}(\phi )\partial_{2}(\psi )\partial_{2}.$$

For σ=(i,j,k), we denote by $\partial^{\sigma }=\partial_{x}^{i}\partial_{1}^{j}\partial_{2}^{k}$. Then, using equation (5.6), we can write

(5.7) $$c(h)=\sum_{\sigma ,\varepsilon }\kappa_{\sigma ,\varepsilon }\partial^{\sigma }(h)\partial^{\varepsilon };\;\;\varepsilon =0,1,\;\;\;\;\text{where}\;\;\kappa_{\sigma ,\varepsilon }=\kappa_{\sigma ,\varepsilon }^{0}+\theta_{1}\kappa_{\sigma ,\varepsilon }^{1}+\theta_{2}\kappa_{\sigma ,\varepsilon }^{2}+\theta_{1}\theta_{2}\kappa_{\sigma ,\varepsilon }^{12}.$$

One obtains

$$\begin{array}{ccc} c(1)\; & =\; & \sum_{\varepsilon }\kappa_{000,\varepsilon }\partial^{\varepsilon },\\ c(x)\; & =\; & \sum_{\varepsilon }(\kappa_{000,\varepsilon }x+\kappa_{100,\varepsilon })\partial^{\varepsilon },\\ c(x^{2})\; & =\; & \sum_{\varepsilon }(\kappa_{000,\varepsilon }x^{2}+2\kappa_{100,\varepsilon }x+2\kappa_{200,\varepsilon })\partial^{\varepsilon },\\ c(\theta_{1})\; & =\; & \sum_{\varepsilon }(\kappa_{000,\varepsilon }\theta_{1}+\kappa_{010,\varepsilon })\partial^{\varepsilon },\\ c(\theta_{2})\; & =\; & \sum_{\varepsilon }(\kappa_{000,\varepsilon }\theta_{2}+\kappa_{001,\varepsilon })\partial^{\varepsilon },\\ c(x\theta_{1})\; & =\; & \sum_{\varepsilon }(\kappa_{000,\varepsilon }x\theta_{1}+\kappa_{100,\varepsilon }\theta_{1}+\kappa_{010,\varepsilon }x+\kappa_{110,\varepsilon })\partial^{\varepsilon },\\ c(\theta_{1}\theta_{2})\; & =\; & \sum_{\varepsilon }(\kappa_{000,\varepsilon }\theta_{1}\theta_{2}+\kappa_{010,\varepsilon }\theta_{2}-\kappa_{001,\varepsilon }\theta_{1}+\kappa_{011,\varepsilon })\partial^{\varepsilon }. \end{array}$$

Let us recall that

$$\begin{array}{cc} \delta (c)(\phi ,\psi )\; & :=L_{\phi }^{\lambda ,\mu }c(\psi )-{\left(-1\right)}^{\left|\phi ||\psi \right|}L_{\psi }^{\lambda ,\mu }c(\phi )-c([\phi ,\psi ])\\ \; & =\partial_{x}c(\psi )-\frac{1}{2}{\left(-1\right)}^{\left|\phi \right|}\left(\eta_{1}(\phi )\eta_{1}(c(\psi ))+\eta_{2}(\phi )\eta_{2}(c(\psi ))\right)\\ \; & \;\;+\mu \partial_{x}\phi c(\psi )-c(\psi )(\phi \partial_{x}-\frac{1}{2}{\left(-1\right)}^{\left|\phi \right|}(\eta_{1}(\phi )\eta_{1}+\eta_{2}(\phi )\eta_{2})+\lambda \partial_{x}\phi )\\ \; & \;\;-{\left(-1\right)}^{\left|\phi ||\psi \right|}\left(\psi \partial_{x}c(\phi )-\frac{1}{2}{\left(-1\right)}^{\left|\psi \right|}(\eta_{1}(\psi )\eta_{1}(c(\phi ))+\eta_{2}(\psi )\eta_{2}(c(\phi ))\right)\\ \; & \;\;+\mu \partial_{x}\psi c(\phi )-c(\phi )(\psi \partial_{x}-\frac{1}{2}{\left(-1\right)}^{\left|\psi \right|}(\eta_{1}(\psi )\eta_{1}+\eta_{2}(\psi )\eta_{2})+\lambda \partial_{x}\psi ))\\ \; & \;\;-c(\phi \partial_{x}c(\psi )-\partial_{x}\phi \psi -\frac{1}{2}{\left(-1\right)}^{\left|\phi \right|}(\eta_{1}(\phi )\eta_{1}(\psi )+\eta_{2}(\phi )\eta_{2}(\psi ))) \end{array}$$

Now, using the terms in *h* in (5.6) for $\left(\phi ,\psi )=(\theta_{2},\theta_{2}\right)$ then for $\left(\phi ,\psi )=(x\theta_{2},\theta_{2}\right)$, we get

$$-\lambda \kappa_{001,001}^{0}+\frac{1}{4}\kappa_{101,000}^{2}=-4\alpha \lambda .$$

Similarly, the terms in $\theta_{1}h$ for $\left(\phi ,\psi )=(\theta_{1},\theta_{1}\right)$ then for $\left(\phi ,\psi )=(x\theta_{1},\theta_{1}\right)$, we get

$$\frac{1}{4}\kappa_{110,000}^{1}-\lambda \kappa_{010,010}^{0}=0.$$

The terms in $\theta_{2}h$ for $\left(\phi ,\psi )=(\theta_{2},\theta_{1}\theta_{2}\right)$ then for $\left(\phi ,\psi )=(x\theta_{2},\theta_{1}\theta_{2}\right)$, we get

$$\lambda \kappa_{001,001}^{0}-\lambda \kappa_{011,001}^{1}-\frac{1}{4}\kappa_{100,000}^{0}-\frac{1}{2}\kappa_{101,000}^{2}+\frac{1}{4}\kappa_{110,000}^{1}=4\alpha \lambda .$$

Consider the terms in $\partial_{1}h$ for $\left(\phi ,\psi )=(\theta_{1},\theta_{1}\theta_{2}\right)$ and $\left(\phi ,\psi )=(x\theta_{1},\theta_{1}\right)$, we obtain

$$-\lambda \kappa_{011,010}^{2}-\lambda \kappa_{010,010}^{1}+\frac{1}{2}\kappa_{110,000}^{1}-\frac{1}{4}\kappa_{101,000}^{2}+\frac{1}{4}\kappa_{100,000}^{0}=0.$$

Consider the terms in $\partial_{1}h$ respectively for $\left(\phi ,\psi )=(\theta_{2},\theta_{1}\theta_{2}\right)$ and for $\left(\phi ,\psi )=(\theta_{1},\theta_{1}\theta_{2}\right)$, we obtain

$$-\frac{1}{2}\kappa_{001,001}^{0}+\frac{1}{2}\kappa_{010,010}^{0}+\frac{1}{4}\kappa_{011,010}^{2}=2\alpha .$$

$$-\frac{1}{2}\kappa_{001,001}^{0}+\frac{1}{4}\kappa_{011,001}^{1}+\frac{1}{2}\kappa_{010,010}^{0}=0.$$

On the other hand, for $\left(\phi ,\psi )=(\theta_{2},\theta_{2}\right)$ and for $\left(\phi ,\psi )=(\theta_{1},\theta_{1}\right)$ consider the terms in $\partial_{x}h$ in (5.6) then we find

$$\frac{1}{4}\kappa_{000,100}^{0}+\frac{1}{2}\kappa_{001,100}^{2}-\frac{3}{2}\kappa_{001,001}^{0}=-4\alpha .$$

$$\frac{1}{4}\kappa_{000,100}^{0}+\frac{1}{2}\kappa_{010,100}^{2}-\frac{3}{2}\kappa_{010,010}^{0}=0.$$

For $\left(f,g)=(\theta_{2},\theta_{1}\theta_{2}\right)$ the terms in $\theta_{1}\partial_{x}h$ give

$$-\frac{3}{4}\kappa_{001,100}^{2}-\frac{1}{4}\kappa_{011,100}^{12}-\frac{1}{2}\kappa_{000,100}^{0}+\frac{3}{4}\kappa_{001,001}^{0}-\frac{3}{4}\kappa_{011,001}^{1}=2\alpha$$

and for $\left(\phi ,\psi )=(\theta_{1},\theta_{1}\theta_{2}\right)$ the terms in $\theta_{2}\partial_{x}h$ imply

$$\frac{3}{4}\kappa_{010,100}^{2}+\frac{1}{4}\kappa_{011,100}^{12}+\frac{1}{2}\kappa_{000,100}^{0}-\frac{3}{4}\kappa_{010,010}^{0}-\frac{3}{4}\kappa_{011,010}^{2}=0.$$

Then, we have the following system:

$$\left\{ \begin{array}{cc} \; & -\lambda \kappa_{001,001}^{0}+\frac{1}{4}\kappa_{101,000}^{2}=-4\alpha \lambda ,\\ \; & \frac{1}{4}\kappa_{110,000}^{1}-\lambda \kappa_{010,010}^{0}=0,\\ \; & \lambda \kappa_{001,001}^{0}-\lambda \kappa_{011,001}^{1}-\frac{1}{4}\kappa_{100,000}^{0}-\frac{1}{2}\kappa_{101,000}^{2}+\frac{1}{4}\kappa_{110,000}^{1}=4\alpha \lambda ,\\ \; & -\lambda \kappa_{011,010}^{2}-\lambda \kappa_{010,010}^{1}+\frac{1}{2}\kappa_{110,000}^{1}-\frac{1}{4}\kappa_{101,000}^{2}+\frac{1}{4}\kappa_{100,000}^{0}=0,\\ \; & -\frac{1}{2}\kappa_{001,001}^{0}+\frac{1}{2}\kappa_{010,010}^{0}+\frac{1}{4}\kappa_{011,010}^{2}=2\alpha ,\\ \; & -\frac{1}{2}\kappa_{001,001}^{0}+\frac{1}{4}\kappa_{011,001}^{1}+\frac{1}{2}\kappa_{010,010}^{0}=0,\\ \; & \frac{1}{4}\kappa_{000,100}^{0}+\frac{1}{2}\kappa_{001,100}^{2}-\frac{3}{2}\kappa_{001,001}^{0}=-4\alpha ,\\ \; & \frac{1}{4}\kappa_{000,100}^{0}+\frac{1}{2}\kappa_{010,100}^{2}-\frac{3}{2}\kappa_{010,010}^{0}=0,\\ \; & -\frac{3}{4}\kappa_{001,100}^{2}-\frac{1}{4}\kappa_{011,100}^{12}-\frac{1}{2}\kappa_{000,100}^{0}+\frac{3}{4}\kappa_{001,001}^{0}-\frac{3}{4}\kappa_{011,001}^{1}=2\alpha ,\\ \; & \frac{3}{4}\kappa_{010,100}^{2}+\frac{1}{4}\kappa_{011,100}^{12}+\frac{1}{2}\kappa_{000,100}^{0}-\frac{3}{4}\kappa_{010,010}^{0}-\frac{3}{4}\kappa_{011,010}^{2}=0. \end{array}\right.$$

Now, for the last system, we substituting the first equation into the second equation, adding the third and the fourth equation, the fifth and the sixth equation, substituting the seventh equation into the eighth equation and adding the ninth equation and the last equation, we yeilds the result α=0. Therefore, we obtain the claim. □

$\underline{\text{Case 2: 2}\beta =m\in N}$. In this case, we have

$$L^{\left(1\right)}=\sum_{k\leq m}t_{\frac{k}{2},\frac{k}{2}}{ϒ}_{\frac{k}{2},\frac{k}{2}}+\sum_{k=1}^{m}t_{-\frac{k}{2},\frac{k}{2}}\Gamma_{-\frac{k}{2},\frac{k}{2}}.$$

Theorem 5.2 *If*2β=m∈N*then the image*$H^{1}\vee H^{1}$*of*$H_{\mathrm{diff}}^{1}\left(\mathrm{osp}(2|2),\mathrm{osp}(1|2);D_{\beta -\frac{k}{2},\beta -\frac{k}{2}}\right)$*under the cup-product is a one-dimensional subspace of*$H_{\mathrm{diff}}^{2}\left(\mathrm{osp}(2|2),\mathrm{osp}(1|2);D_{\beta -\frac{k}{2},\beta -\frac{k}{2}}\right)$*spanned by*

$$\Omega_{-\frac{k}{2},\frac{k}{2}}^{1}={ϒ}_{\frac{k}{2},\frac{k}{2}}\vee \Gamma_{-\frac{k}{2},\frac{k}{2}}.$$

Proof In this case, the space $H^{1}\vee H^{1}$ is generated by the two cup-products:

$$\Omega_{-\frac{k}{2},\frac{k}{2}}^{1}={ϒ}_{\frac{k}{2},\frac{k}{2}}\vee \Gamma_{-\frac{k}{2},\frac{k}{2}}\;\;\text{and}\;\;\Omega_{-\frac{k}{2},\frac{k}{2}}^{2}={ϒ}_{-\frac{k}{2},\frac{k}{2}}\vee \Gamma_{-\frac{k}{2},-\frac{k}{2}}.$$

Using simple computations, we confirm that

$$\Omega_{-\frac{k}{2},\frac{k}{2}}^{2}=-\Omega_{-\frac{k}{2},\frac{k}{2}}^{1}$$

where

$$\Omega_{-\frac{k}{2},\frac{k}{2}}^{1}(\phi ,\psi )={ϒ}_{\frac{k}{2},\frac{k}{2}}\vee \Gamma_{-\frac{k}{2},\frac{k}{2}}(\phi ,\psi )={\left(-1\right)}^{\left|\phi ||\Gamma_{-\frac{k}{2},\frac{k}{2}}\right|}[{ϒ}_{\frac{k}{2},\frac{k}{2}}(\phi ),\Gamma_{-\frac{k}{2},\frac{k}{2}}(\psi )]+{\left(-1\right)}^{\left|{ϒ}_{\frac{k}{2},\frac{k}{2}}|(|\phi |+|\Gamma_{-\frac{k}{2},\frac{k}{2}}|\right)}[\Gamma_{-\frac{k}{2},\frac{k}{2}}(\phi ),{ϒ}_{\frac{k}{2},\frac{k}{2}}(\psi )]=[{ϒ}_{\frac{k}{2},\frac{k}{2}}(\phi ),\Gamma_{-\frac{k}{2},\frac{k}{2}}(\psi )]+[\Gamma_{-\frac{k}{2},\frac{k}{2}}(\phi ),{ϒ}_{\frac{k}{2},\frac{k}{2}}(\psi )]=\left[k\eta_{1}(\partial_{2}(\phi )),k\eta_{1}(\partial_{2}(\psi ))\eta_{1}\eta_{2}^{2k-1}-{\left(-1\right)}^{\left|\psi \right|}\left(\partial_{2}(\psi )\eta_{2}^{2k+1}-\eta_{1}(\theta_{2}\partial_{2}(\psi ))\eta_{1}^{2k+1}\right)\right]\;\;-{\left(-1\right)}^{\left|\phi \right|}\left[\partial_{2}(\phi )\eta_{1}+\theta_{2}\eta_{2}\eta_{1}(\phi )\eta_{2},k\eta_{1}(\partial_{2}(\psi ))\eta_{1}\eta_{2}^{2k-1}\right]\;\;+{\left(-1\right)}^{\left|\phi |+|\psi \right|}\left[\partial_{2}(\phi )\eta_{1}+\theta_{2}\eta_{2}\eta_{1}(\phi )\eta_{2},\partial_{2}(\psi )\eta_{2}^{2k+1}-\eta_{1}(\theta_{2}\partial_{2}(\psi ))\eta_{1}^{2k+1}\right]\;\;+\left[k\eta_{1}(\partial_{2}(\phi ))\eta_{1}\eta_{2}^{2k-1},k\eta_{1}(\partial_{2}(\psi ))-{\left(-1\right)}^{\left|\psi \right|}\left(\partial_{2}(\psi )\eta_{1}+\theta_{2}\eta_{2}\eta_{1}(\psi )\eta_{2})\right)\right]\;\;-{\left(-1\right)}^{\left|\phi \right|}\left[\partial_{2}(\phi )\eta_{2}^{2k+1}-\eta_{1}(\theta_{2}\partial_{2}(\phi ))\eta_{1}^{2k+1},k\eta_{1}(\partial_{2}(\psi ))\right]\;\;+{\left(-1\right)}^{\left|\phi |+|\psi \right|}\left[\partial_{2}(\phi )\eta_{2}^{2k+1}-\eta_{1}(\theta_{2}\partial_{2}(\phi ))\eta_{1}^{2k+1},\partial_{2}(\psi )\eta_{1}+\theta_{2}\eta_{2}\eta_{1}(\psi )\eta_{2})\right]={\left(-1\right)}^{|\psi |+k}\left({\left(-1\right)}^{\left|\phi \right|}\partial_{2}(\phi )\eta_{1}\partial_{2}(\psi )-\eta_{1}\partial_{2}(\phi )\partial_{2}(\psi )\right)\partial_{x}^{k}\partial_{2}\;\;\;+{\left(-1\right)}^{|\psi |+k}k\left(\theta_{1}\partial_{2}(\phi )\partial_{x}\partial_{2}(\psi )+k\theta_{1}\partial_{x}\partial_{2}(\phi )\partial_{2}(\psi )\right)\partial_{x}^{k}\partial_{2}\;\;\;+{\left(-1\right)}^{|\psi |+k}\left(\theta_{1}\partial_{2}(\phi )\partial_{2}(\psi )\partial_{x}^{k+1}\partial_{2}-\theta_{2}\partial_{2}(\phi )\partial_{2}(\psi )\partial_{x}\partial_{1}\right)\;\;-(k+1)\left(\theta_{2}\partial_{2}(\phi )\partial_{x}\partial_{2}(\psi )+\theta_{2}\partial_{x}\partial_{2}(\phi )\partial_{2}(\psi )\right)\partial_{x}^{k}\partial_{1}\;\;\;+{\left(-1\right)}^{k}\theta_{2}\left(\partial_{2}(\phi )\partial_{1}\partial_{2}(\psi )-{\left(-1\right)}^{\left|\phi \right|}\partial_{1}\partial_{2}(\phi )\partial_{2}(\psi )\right)\partial_{x}^{k+1}\;\;\;-{\left(-1\right)}^{|\phi |+k}(k+2)\theta_{1}\theta_{2}\left(\partial_{2}(\phi )\partial_{x}\partial_{2}(\psi )+\partial_{x}\partial_{2}(\phi )\partial_{2}(\psi )\right)\partial_{x}^{k+1}\;\;\;-2{\left(-1\right)}^{|\phi |+k}\left(\partial_{2}(\phi )\partial_{2}(\psi )\partial_{1}\partial_{2}+\theta_{1}\theta_{2}\partial_{2}(\phi )\partial_{2}(\psi )\partial_{x}^{2}\right)\;\;-{\left(-1\right)}^{|\phi |+k}k\left(\partial_{2}(\phi )\partial_{x}\partial_{2}(\psi )+\partial_{x}\partial_{2}(\phi )\partial_{2}(\psi )\right)\partial_{x}^{k-1}\partial_{1}\partial_{2}.$$

The proof is almost the same as the one for the previous theorem. Indeed, we must demonstrate that $\Omega_{-\frac{k}{2},\frac{k}{2}}^{1}$ is a non-trivial osp(1|2)-relative 2-cocycle. That is, the equation

(5.8) $$\tau .\Omega_{-\frac{k}{2},\frac{k}{2}}^{1}=\delta (c),$$

has a solution if and only if τ=0. We can certainly write *c* as in (5.7).

Here, we simply provide the parameters of identifications that allow us to get the result. For (5.8), considering the terms in $\partial_{x}^{k}\partial_{2}$, for $\left(\phi ,\psi )=(\theta_{2},\theta_{1}\theta_{2}\right)$, the terms in $\partial_{x}^{k}\partial_{1}$, for $\left(\phi ,\psi )=(\theta_{1},\theta_{1}\theta_{2}\right)$, the terms in $\partial_{x}^{k-1}\partial_{1}\partial_{2}$, for $\left(\phi ,\psi )=(x\theta_{2},\theta_{1}\theta_{2}\right)$, $\left(x\theta_{1},\theta_{1}\theta_{2}\right)$, $\left(x\theta_{2},\theta_{2}\right)$, $\left(x\theta_{1},\theta_{1}\right)$ and the terms in $\partial_{x}^{k}\partial_{1}\partial_{2}$, for $\left(f,g)=(\theta_{2},\theta_{1}\theta_{2}\right)$, $\left(\theta_{1},\theta_{1}\theta_{2}\right)$, $\left(\theta_{2},\theta_{2}\right)$, $\left(\theta_{1},\theta_{1}\right)$, then we have

$$\kappa_{001,k10}^{0}+\kappa_{010,k01}^{0}=-{\left(-1\right)}^{k}\frac{2}{3}\tau ,$$

and

$$\kappa_{010,k01}^{0}+\kappa_{001,k10}^{0}=-{\left(-1\right)}^{k}\tau .$$

Then τ=0. □

The second differential osp(1|2)-relative cohomology space of osp(2|2) with coefficients in $D_{\lambda ,\mu }^{2}$ ‘‘$H_{\mathrm{diff}}^{2}(\mathrm{osp}(2|2),\mathrm{osp}(1|2);D_{\lambda ,\mu }^{2})$” is the subject of the next conjecture.

Conjecture 5.1 *One has*

$$H_{\mathrm{diff}}^{1}\left(\mathrm{osp}(2|2),\mathrm{osp}(1|2);D_{\lambda ,\mu }^{2}\right)\vee H_{\mathrm{diff}}^{1}\left(\mathrm{osp}(2|2),\mathrm{osp}(1|2);D_{\lambda ,\mu }^{2}\right)=H_{\mathrm{diff}}^{2}\left(\mathrm{osp}(2|2),\mathrm{osp}(1|2);D_{\lambda ,\mu }^{2}\right).$$

In computing second cohomology spaces, which are often challenging to derive, the conjecture is a key unresolved topic.

## 6 Integrability conditions

The cup products of first cohomology classes are used to describe the obstructions “conditions” under which an infinitesimal deformation ensures the existence of a formal deformation. The second cohomology space $H_{\mathrm{diff}}^{2}\left(\mathrm{osp}(2|2),\mathrm{osp}(1|2);D_{\lambda ,\mu }^{2}\right)$ has these obstructions.

We consider an infinitesimal deformation

$$\overset{˜}{L}=L+L^{1}$$

of the natural action L of osp(2|2) on the space $\mathrm{End}(S_{\beta }^{2})$ and we investigate the necessary and sufficient conditions to extend it to a formal one:

$$\overset{˜}{L}=L+L^{1}+\sum_{i}Q_{i}^{2}L_{i}^{2}+\sum_{i}Q_{i}^{3}L_{i}^{3}+\cdots ,$$

where

$$L^{1}=\left\{ \begin{array}{ccc} \sum_{k\geq 0}t_{\beta -\frac{k}{2},\beta -\frac{k}{2}}\omega_{\beta -\frac{k}{2},\beta -\frac{k}{2}}\; & \text{if}\; & 2\beta \notin N\\ \sum_{k\leq m}t_{\frac{k}{2},\frac{k}{2}}{ϒ}_{\frac{k}{2},\frac{k}{2}}+\sum_{k=1}^{m}t_{\frac{k}{2},\frac{k}{2}}\Gamma_{-\frac{k}{2},\frac{k}{2}}\; & \text{if}\; & 2\beta =m\in N \end{array}\right.$$

and the terms $L_{i}^{2}$, $L_{i}^{3}\cdots$ are linear maps from osp(2|2) to $\mathrm{End}(S_{\beta }^{2})$ such that the map

$$\overset{˜}{L}:\mathrm{osp}(2|2)⟶C[[t_{\beta -\frac{k}{2},\beta -\frac{k}{2}},t_{\frac{k}{2},\frac{k}{2}},t_{-\frac{k}{2},\frac{k}{2}}]]⊗\mathrm{End}(S_{\beta }^{2}),$$

satisfies the homomorphism requirement ‘‘condition” (4.3). $Q_{i}^{j}$ are monomial in the parameters $t_{\beta -\frac{k}{2},\beta -\frac{k}{2}}$, $t_{\frac{k}{2},\frac{k}{2}}$, $t_{-\frac{k}{2},\frac{k}{2}}$ with degree *j* and with the same parity of $L_{i}^{j}$.

Our main theorems are listed below, we must distinguish two cases.

$\underline{\text{Case 1: 2}\beta \notin N}$. In this case, we have $L^{1}=\sum_{k\geq 0}t_{\beta -\frac{k}{2},\beta -\frac{k}{2}}\omega_{\beta -\frac{k}{2},\beta -\frac{k}{2}}$.

Theorem 6.1 *The conditions*

(6.9) $$t_{\beta -\frac{k}{2},\beta -\frac{k}{2}}=0,\;\;\text{for all}\;\;k\geq 0$$

*are necessary and sufficient for the integrability of the infinitesimal deformation*(4.2)*.*

*In addition, any formal deformation is equivalent to its infinitesimal part.*

Proof For the second-order terms, the condition (4.3) yields the following equation:

(6.10) $$\delta L^{2}=\frac{1}{2}\sum_{k\geq 0}t_{\beta -\frac{k}{2},\beta -\frac{k}{2}}^{2}\Omega_{\beta -\frac{k}{2},\beta -\frac{k}{2}}.$$

It follows that, the right hand side of (6.10) must be a coboundary. However, according to Theorem 5.1, $\Omega_{\beta -\frac{k}{2},\beta -\frac{k}{2}}$ is a non-trivial osp(1|2)-relative 2-cocycle, therefore $t_{\beta -\frac{k}{2},\beta -\frac{k}{2}}^{2}=0$ for all k≥0. Thus, the condition (6.9) is necessary.

We will explicitly find a deformation of $L_{X}$ whenever conditions (6.9) are satisfied to demonstrate that conditions (6.9) are sufficient. It is possible to select zero for solution $L^{\left(2\right)}$ of (6.10). It is evident that a deformation (which is of order 1 in t) is obtained by selecting the highest-order terms $L^{\left(m\right)}$ with m≥3, which are also identically zero.

The solutions $L^{k}$ of the Maurer-Cartan equations (4.5) are defined up to a 1-cocycle and it has been proved in works [1], [14] that different choices of solutions correspond to equivalent deformations. As a result we can always reduce $L^{k}$, for k=2 to zero using equivalence. The highest-order terms $L^{k}$ with k≥3, then satisfy the equation $\delta (L^{k})$ and can be reduced to the identically zero map, via recurrence. □

$\underline{\text{Case 2: 2}\beta =m\in N}$. In this case, we have

$$L^{\left(1\right)}=\sum_{k\leq m}t_{\frac{k}{2},\frac{k}{2}}{ϒ}_{\frac{k}{2},\frac{k}{2}}+\sum_{k=1}^{m}t_{-\frac{k}{2},\frac{k}{2}}\Gamma_{-\frac{k}{2},\frac{k}{2}}.$$

Theorem 6.2 *The conditions*

$$t_{\frac{k}{2},\frac{k}{2}}t_{-\frac{k}{2},\frac{k}{2}}-t_{-\frac{k}{2},\frac{k}{2}}t_{-\frac{k}{2},-\frac{k}{2}}=0,\;\;\text{for all}\;\;k\geq 0$$

*are necessary and sufficient for the integrability of the infinitesimal deformation*(4.2)*.*

*Moreover, any formal deformation is equivalent to its infinitesimal part.*

These factors provide a classification of the formal deformations, similar to the first case.

Proof In this case, the equation (4.3) can be expressed as follows

$$\delta L^{2}=\frac{1}{2}\sum_{k\geq 0}(t_{\frac{k}{2},\frac{k}{2}}t_{-\frac{k}{2},\frac{k}{2}}-t_{-\frac{k}{2},\frac{k}{2}}t_{-\frac{k}{2},-\frac{k}{2}})\Omega_{-\frac{k}{2},\frac{k}{2}}^{1}.$$

The fact that the map $\Omega_{-\frac{k}{2},\frac{k}{2}}^{1}$ is non-trivial osp(1|2)-relative 2-cocycle determines the second order integrability conditions as shown in Theorem 5.2. As mentionel above, the conditions are sufficient, and the terms $L^{k}$ with k≥2 can be chosen to be identically zero. □

Example 6.1 Consider 2β∉N, let $t_{\beta -\frac{k}{2},\beta -\frac{k}{2}}=0$, k∈Z. Consequently, the following deformation of $S_{\beta }^{2}$ is obtained with two families of independent parameters

$${\overset{˜}{L}}_{f}=L_{f}.$$

Example 6.2 Consider 2β=m∈N, let $t_{\frac{k}{2},\frac{k}{2}}=t_{-\frac{k}{2},\frac{k}{2}}=t$, k∈Z. Consequently, the following deformation of $S_{\beta }^{2}$ is obtained with two families of independent parameters

$${\overset{˜}{L}}_{f}=L_{f}+\sum_{k\leq m}t{ϒ}_{\frac{k}{2},\frac{k}{2}}+\sum_{k=1}^{m}t\Gamma_{-\frac{k}{2},\frac{k}{2}}.$$

Example 6.3 Consider 2β=m∈N, let $t_{\frac{k}{2},\frac{k}{2}}=\frac{t}{2}$ and $t_{-\frac{k}{2},\frac{k}{2}}=0$, k∈Z. Consequently, the following deformation of $S_{\beta }^{2}$ is obtained with two families of independent parameters

$${\overset{˜}{L}}_{f}=L_{f}+\frac{1}{2}\sum_{k\leq m}t{ϒ}_{\frac{k}{2},\frac{k}{2}}.$$

Example 6.4 Consider 2β=m∈N, let $t_{-\frac{k}{2},\frac{k}{2}}=\frac{t}{3}$ and $t_{\frac{k}{2},\frac{k}{2}}=0$, k∈Z. Consequently, the following deformation of $S_{\beta }^{2}$ is obtained with two families of independent parameters

$${\overset{˜}{L}}_{f}=L_{f}+\frac{1}{3}\sum_{k=1}^{m}t\Gamma_{-\frac{k}{2},\frac{k}{2}}.$$

## Ethics approval and consent to participate

All authors approve ethics and consent to participate.

## Consent for publication

The authors declare the consent for manuscript publication.

## Funding

This research project was funded by the 10.13039/501100022230Deanship of Scientific Research, Princess Nourah Bint Abdulrahman University, through the Program of Research Project Funding After Publication, grant No (43-PRFA-P-80).

## CRediT authorship contribution statement

**Areej A Almoneef:** Writing – review & editing, Resources, Methodology, Funding acquisition, Data curation. **Meher Abdaoui:** Writing – original draft, Visualization, Resources, Project administration, Formal analysis, Data curation, Conceptualization. **Abderraouf Ghallabi:** Visualization, Resources, Methodology, Formal analysis, Data curation.

## Declaration of Competing Interest

The authors declare that they have no known competing financial interests or personal relationships that could have appeared to influence the work reported in this paper.

## Data Availability

Data sharing not applicable to this paper as no datasets were generated or analysed during this research work.
